# Interface‐Directed High Fluorescence Efficiency Two‐Dimensional Molecular Crystals With Surface‐Exposed Active Sites

**DOI:** 10.1002/advs.76473

**Published:** 2026-07-09

**Authors:** Shuya Liu, Yueqiang Zhang, Chuanqin Cheng, Jin Wang, Feiyong Chen, Yonglei Wang, Yanjun Gong

**Affiliations:** ^1^ Institute of Resources and Environment Innovation Shandong Jianzhu University No. 1000 Fengming Road Jinan Shandong P. R. China; ^2^ School of Municipal and Environmental Engineering Shandong Jianzhu University No. 1000 Fengming Road Jinan Shandong P. R. China; ^3^ School of Chemistry and Chemical Engineering Shandong University Jinan Shandong P. R. China; ^4^ Department of Chemistry Key Laboratory of Organic Optoelectronics and Molecular Engineering of Ministry of Education Tsinghua University Beijing P. R. China

**Keywords:** chalcogen bonding, crystal engineering, dimethyl sulfide (DMS), fluorescence sensing, two‐dimensional (2D) molecular crystals

## Abstract

Two‐dimensional (2D) molecular crystals offer significant advantages for many applications due to their ultrahigh surface‐to‐volume ratio. Nonetheless, controlling the oriented growth of 2D crystals to expose more active sites remains a challenge. Herein, we develop a strategy that combines molecular design with interfacial assembly to achieve the oriented formation of 2D crystals of **Me‐FBSe**. Uniquely, this architecture preserves the high photoluminescence quantum yield intrinsic to the crystalline state, while simultaneously ensuring the high‐density surface exposure of active sites. The integration of high luminescence efficiency and accessible active sites enables ultrasensitive detection of dimethyl sulfide (DMS) vapor at 50 ppb. Notably, this sensing material can detect DMS in aqueous environments via headspace vapor analysis, with a minimum detectable concentration of 100  ppb, demonstrating its reliability in practical monitoring applications. This work establishes a new strategy for the directional exposure of functional groups in high‐performance sensors.

## Graphical abstract

Here, we designed the **Me‐FBSe** molecule and prepared a two‐dimensional molecular crystal via liquid‐liquid interface assembly. The crystal preserves the high photoluminescence quantum while simultaneously ensuring the high‐density surface exposure of active sites. This synergistic effect ultimately enables ultra‐sensitive detection of dimethyl sulfide vapor, with a detection limit of 50 ppb.

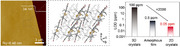

## 1 Introduction

Two‐dimensional materials‐including graphene, molybdenum disulfide (MoS_2_), two‐dimensional molecular crystals (2DMCs), and graphitic carbon nitride (g‑C_3_N_4_)‐have attracted intense attention for applications in catalysis, sensing, electronics, and energy conversion [1, 2, 3, 4, 5, 6, 7, 8, 9, 10, 11, 12]. Their thin geometries confer ultrahigh surface‐area‐to‐volume ratios and abundant active sites, while the in‐plane atomic or molecular stacking endow them with unique physical and chemical properties. In particular, 2DMCs assembled from small organic molecules or polymers via noncovalent interactions, form ultrathin, ordered lattices with abundant surface‐accessible functional sites, improving sensing, catalysis, and device performance [13, 14, 15, 16]. However, conventional solution‐phase assembly often thermodynamically favored three‐dimensional (3D), close‐packed crystals that buries recognition motifs. Therefore, directing growth into strictly two dimensions and selectively exposing functional facets is essential to realize the potential of 2DMCs. Bottom‐up interfacial growth on liquid‐liquid or liquid‐air templates, combined with solution‐shearing and meniscus‐guided coating, promotes anisotropic nucleation, suppresses out‐of‐plane growth, and aligns conjugated molecules into extended lateral single‐crystal domains [17, 18, 19, 20, 21, 22, 23, 24, 25, 26].

Fluorescent sensors based on π‐conjugated organic layers have been widely explored for trace detection of explosives, narcotics, and environmental pollutants [27, 28, 29, 30, 31, 32, 33, 34, 35, 36, 37, 38, 39, 40]. In these sensors, analyte vapors interact directly with the solid‐state emitter, enabling rapid photoluminescence quenching or wavelength shifts via gas‐solid interfacial recognition. Crystalline thin films possess well‐defined molecular packing and long‐range order, which can provide uniform diffusion pathways and structurally consistent recognition environments for guest molecules. These characteristics are advantageous for understanding and controlling analyte transport and sensing behavior compared with amorphous films, whose local structures are often more heterogeneous [41, 42]. Unfortunately, many fluorescent molecules thermodynamically pack into 3D lattices via *π–π* stacking or hydrogen bonding, which buries recognition motifs and forces analytes to diffuse into the bulk, slowing response and degrading sensitivity. Consequently, rational control of assembly geometry, to produce layered crystals that present target binding sites (e.g., nitroaromatic cavities, coordination pockets, hydrogen‐bond donors) at the surface, is essential. Although interface‐directed synthesis and solution processing now deliver large‐area organic single crystals, reliable strategies to lock functional groups at exposed facets are still lacking. Addressing this molecular‐interface design challenge will enable 2DMCs that combine crystalline stability with highly accessible active sites, thereby enhancing the sensitivity and selectivity of thin‐film fluorescent sensors.

To address this challenge, we developed a donor‐acceptor‐donor (D‐A‐D) fluorophore, **Me‐FBSe**, that integrates high photoluminescence efficiency, molecular recognition, and interfacial ordering capability. The molecular architecture was conceived to couple radiative efficiency with selective chalcogen‐bond recognition and directional crystal growth at liquid interfaces, enabling 2D fluorescent films with exposed active sites. Using this design, we prepared centimeter‐scale, thickness‐tunable 2D molecular crystals in which benzoselenadiazole moieties are preferentially presented at the surface. The films exhibit high quantum yields, attributed to cluster‐trapped excited states within the ordered lattice, and deliver highly selective, ultrasensitive detection of dimethyl sulfide down to low‐ppb levels. This integrated strategy of photophysics‐guided chromophore design combined with interface‐directed assembly provides a promising strategy for constructing crystalline fluorescent films with high fluorescence efficiency and highly accessible sensing sites for chemical sensing.

## 2 Results and Discussion

### 2.1 Molecular Design

**FIGURE 1:**
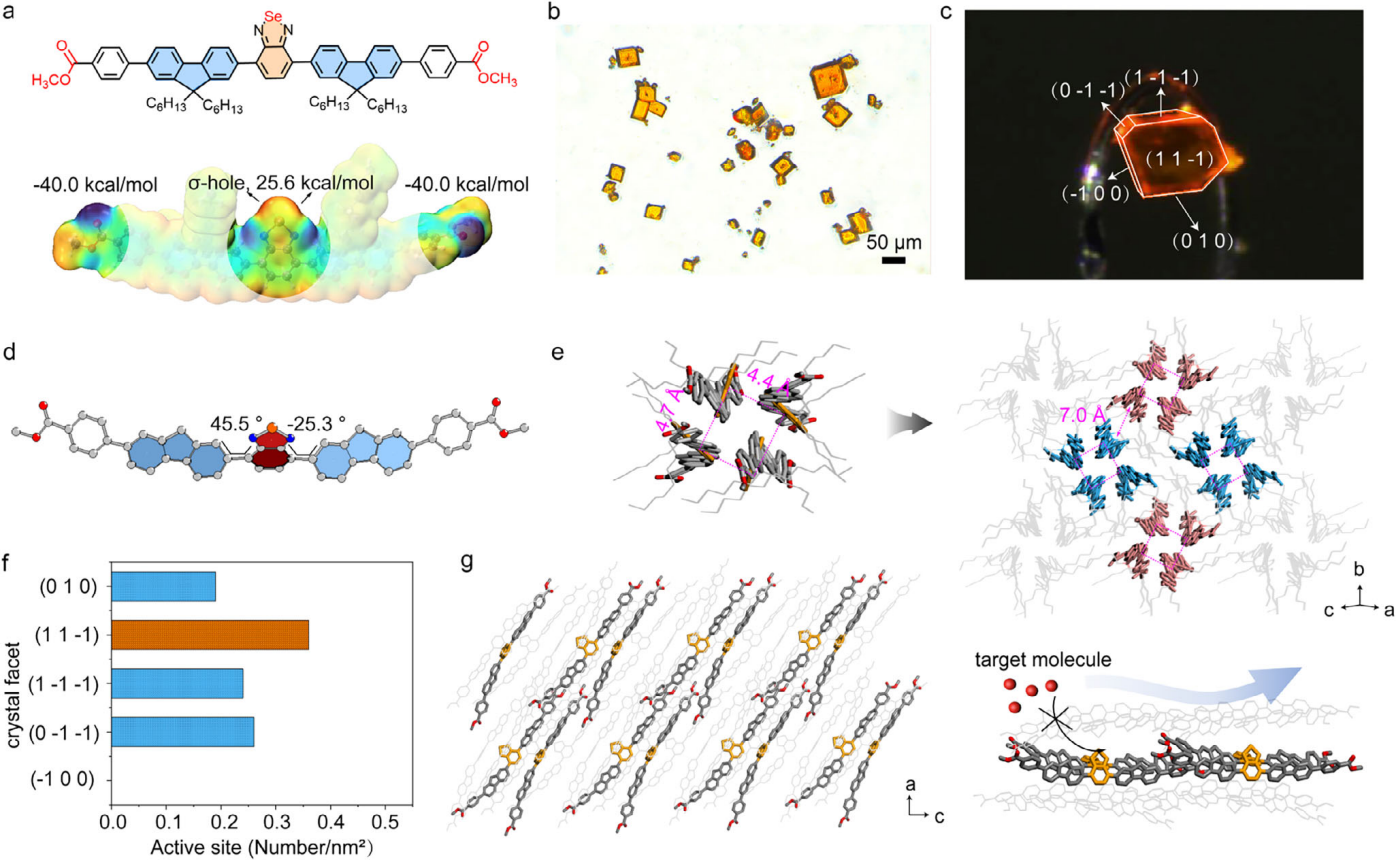
The 3D structures of fluorophore **Me‐FBSe** crystals. (a) Molecular structure and ESP of **Me‐FBSe**. (b) Optical micrograph of **Me‐FBSe** crystals. (c) Single crystal orientation photograph of the **Me‐FBSe** crystal. (d) The twist structure of **Me‐FBSe**, H atoms and alkyl groups were removed from single crystal for clarification. (e) A view of a tetrameric clusters and quadrilateral array of the crystal packing in **Me‐FBSe**, H atoms were removed for clarification. (f) Density of exposed active sites on different crystal facets of **Me‐FBSe**. (g) Simplified crystal packing diagram of **Me‐FBSe** (top and side views). H atoms and alkyl groups were removed for clarity to reveal the shielding of internal active sites.

Here, a D‐A‐D structure fluorophore, **Me‐FBSe** (Figure 1a) was designed, featuring a benzoselenadiazole electron‐accepting core flanked by two fluorene‐based donors and terminated with polar methyl ester groups. Detailed synthesis and characterization of this molecule are provided in the supporting information (Scheme S1, Figures S1–S13). Theoretical calculations confirm that this pronounced push‐pull architecture generates a significant intramolecular charge transfer effect that stabilizes the hybridized localized‐charge transfer (HLCT) excited state, laying the foundation for efficient luminescence (Figure S14) [43, 44]. As shown by its molecular electrostatic potential (ESP) mapping (Figure 1a), the selenium atom exhibits a distinct σ‐hole with a positive ESP value of +25.6 kcal/mol, indicating a potential binding site for chalcogen bonding [45]. Furthermore, the polar methyl ester termini, characterized by negative ESP values, enhance the molecular polarity and are anticipated to facilitate favorable solid‐state packing.

### 2.2 Me‐FBSe Crystal Structures

The self‐assembly behavior of **Me‐FBSe** was first studied using a liquid‐phase strategy based on poor solvent precipitation. Specifically, 1 mL of ethanol was added to 100 µL of a chloroform solution of **Me‐FBSe** (0.8 mM), and the resulting mixture was aged for 7 days, yielding 3D cubic block‐shaped crystals. The crystals were characterized by optical microscopy (Figure 1b) and scanning electron microscopy (SEM) (Figure S15), showing well‐defined morphologies with dimensions in the micrometer range. To gain a better understanding of the structure and packing of **Me‐FBSe**, single‐crystal XRD analysis was performed. The crystallizes in the monoclinic **P2_1_/c** space group with lattice constants a  =  23.4224(11) Å, b  =  23.2391(10) Å and c  =  23.2876(10) Å (Table S1). As shown in Figure 1c, a crystal orientation analysis of the obtained 3D crystal reveals multiple well‐defined facets, concluding (0 ‐1 ‐1) (1 ‐1 ‐1) (1 1 ‐1) (‐1 0 0) (010). The single‐crystal structure reveals that the **Me‐FBSe** adopts a twisted conformation in the solid state, with dihedral angles of 25.3° and 45.5° between the D and A units (Figure 1d). This twisted molecular structure is crucial for facilitating the HLCT excited state, which contributes to the high fluorescence efficiency. The twisted molecules of **Me‐FBSe** first assemble into tetrameric clusters, primarily stabilized by van der Waals interactions. Within these clusters, the benzoselenadiazole active sites and alkyl chains are oriented outward. A view along the (111) facet (Figure 1e) reveals that these clusters are organized into a quadrilateral array. Notably, adjacent clusters within this array maintain a spatial separation for D‐A‐D units of approximately 7 Å, which is mediated by hydrophobic‐hydrophobic interactions between the outward‐oriented alkyl chains. Then, analysis of the (11‐1) plane (Figure S16) reveals the directional forces that guide the long‐range molecular order C─H···O (3.245 Å) interactions facilitate molecular alignment along the <101> direction. Crystallographic mapping shows a stark contrast between different facets: the (11‐1) facet possesses the highest density of exposed active sites, while the (‐1 0 0) facet completely buries them (Figure S17 and Figure 1f). As shown in Figure 1g, the dense packing of **Me‐FBSe** 3D crystals results in the burial of the active benzoselenadiazole motifs within the crystal interior, thereby rendering the active sites in the bulk crystal largely inaccessible to target molecules. Overall, **Me‐FBSe** exhibits a strong propensity for 3D crystallization driven by weak intermolecular interactions such as electrostatic interaction and hydrogen bonding. This growth habit leads to the formation of bulk crystals wherein the majority of active sites remain embedded within the structure, greatly limiting their efficiency in catalytic and sensing applications.

### 2.3 Fabrication and Structural Characterization of 2D Crystals of Me‐FBSe

**FIGURE 2:**
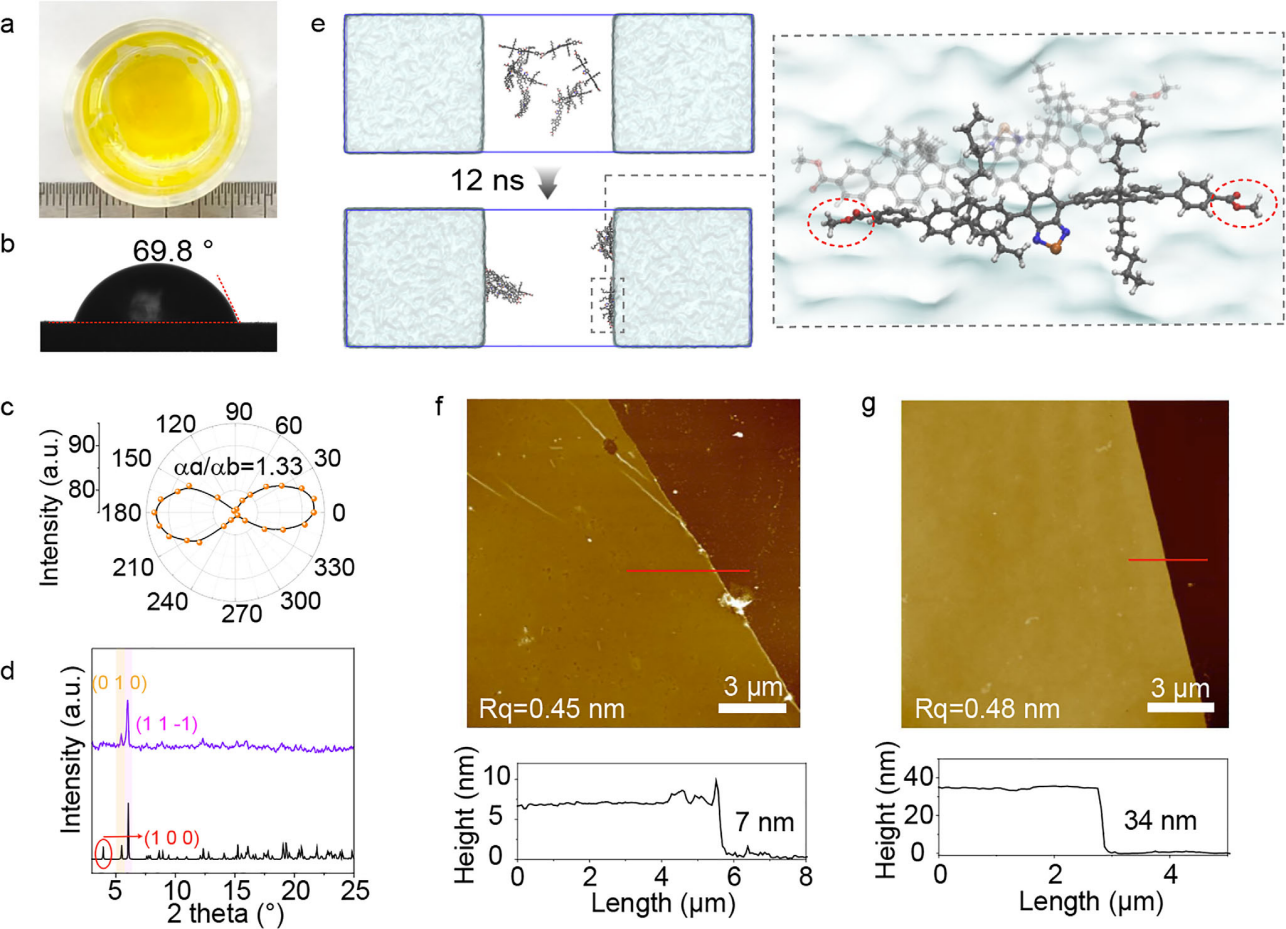
Structural characterization and morphology of 2D crystals of **Me‐FBSe**. (a) The macro photograph, (b) water contact angle measurements, and (c) polarized emissions of 2D crystals of **Me‐FBSe**. (d) XRD patterns of 2D crystals of **Me‐FBSe** (top) and single crystal of **Me‐FBSe** (below). (e) Snapshots of molecular dynamics (MD) trajectory frames from 0 ns to 12 ns illustrating the **Me‐FBSe** lateral packing at the vacuum‐water interface. AFM height image along with the corresponding height profile of 2D crystals assembled by 2 mg mL^−1^ (f) and 5 mg mL^−1^ (g) **Me‐FBSe** at the chloroform‐water interface.

To overcome the challenge of poor active site accessibility, an oil/water interfacial assembly method was used to steer molecular packing toward lateral growth, ultimately yielding ultrathin two‐dimensional architectures. Typically, a 100 µL chloroform solution of **Me‐FBSe** at a specific concentration was drop‐cast onto a 3 mL water subphase contained in a 5 mL vial (2 cm in diameter) maintained at 4°C to slow the evaporation of chloroform. After complete evaporation of the solvent, a centimeter‐scale film, 2D crystals of **Me‐FBSe**, formed on the air/water interface (Figure 2a), which could be readily transferred onto other substrates, showing smooth and uniform (Figure S18). The observed water contact angle of 2D crystals of **Me‐FBSe** is 69.8° (Figure 2b), suggesting a preferentially lateral molecular arrangement that may expose more terminal hydrophilic carbonyl groups. This hydrophilic adhesion means the 2D crystals of **Me‐FBSe** can be easily integrated onto substrates of various materials, including flexible or rigid ones.

Polarized fluorescent microscope images were captured at different polarization angles to investigate the crystallinity of the 2D crystals of **Me‐FBSe**. As depicted in Figure 2c, the fluorescence intensity exhibited an initial decline as the angle increased from 0° to 90°, followed by a gradual recovery from 90° to 180°. This periodic variation pattern repeated consistently over the subsequent angular ranges of 180°–270° and 270°–360°, which suggests that the 2D crystals of **Me‐FBSe** had highly ordered crystallographic alignment. Based on the comparative analysis between the experimental XRD of 2D crystals of **Me‐FBSe** and the simulated XRD of single‐crystal (Figure 2d), it was found that the 2D crystals selectively exposes the (1 1 ‐1) crystal facet, which possesses the highest abundance of active sites for benzoselenadiazole. The selected area electron diffraction (SAED) result further demonstrates (Figure S19) the single‐crystalline nature of the 2D crystals of **Me‐FBSe**.

Building upon these experimental findings, theoretical calculations and molecular dynamics (MD) simulations were employed to demonstrate the underlying mechanism of **Me‐FBSe** molecular orientation, specifically the directive role of water molecules. Squared charge population analysis (SCPA) charge population analysis (Figure S20) reveals that the carbonyl oxygen atoms at both termini of the **Me‐FBSe** carry substantial negative charges (−0.42), while hydrogen atoms of water molecules exhibit positive charges (+0.09) [46]. This complementary charge enables water molecules to act as dynamic structural templates, forming directional hydrogen bonds with polar carbonyl and ether oxygen atoms in the terminal ester groups (e.g., C═O⋯H─O─H). MD simulation snapshots illustrating the time‐dependent self‐assembly process of **Me‐FBSe** molecules at the water‐vacuum‐water interface (Figure 2e). The image shows the transformation from initially dispersed **Me‐FBSe** molecules (0 ns) in the vacuum to their complete aggregation and adsorption at the water interface after 12 ns. Notably, the **Me‐FBSe** molecules adopt a predominantly lateral configuration on the water surface, consistent with the water‐mediated strategy designed to steer packing toward two‐dimensional growth. The interface simultaneously anchors both **Me‐FBSe** molecular termini, not only enforcing a lying‐down molecular orientation but also providing thermodynamic driving force for in‐plane expansion of the two‐dimensional lattice, ultimately guiding preferential crystal growth along specific crystallographic orientations. The synergy between interfacial confinement and water‐directed hydrogen bonding promotes two‐dimensional nucleation while suppressing volumetric thickening, resulting in 2D crystals with maximally exposed active sites.

Importantly, atomic force microscopy (AFM) images showed the great homogeneity of the as‐prepared 2D **Me‐FBSe** crystals and the thickness of the 2D **Me‐FBSe** crystals could be controlled from several to tens of nanometers by changing the concentration of the **Me‐FBSe** chloroform solution ranging from 2 to 10 mg mL^−1^(Figure 2f,g, and Figure S21). Specifically, a concentration of 2 mg mL^−1^ resulted in an ultrathin 7 nm film with an extremely low root‐mean‐square (RMS) roughness of less than 0.45 nm (Figure 2f), while 5 mg mL^−1^ yielded a 34 nm film (Figure 2g). Hence, this simple strategy may provide a useful approach for constructing oriented 2D molecular crystals with preferential facet exposure.

### 2.4 Excited‐State Analysis of Me‐FBSe

**FIGURE 3:**
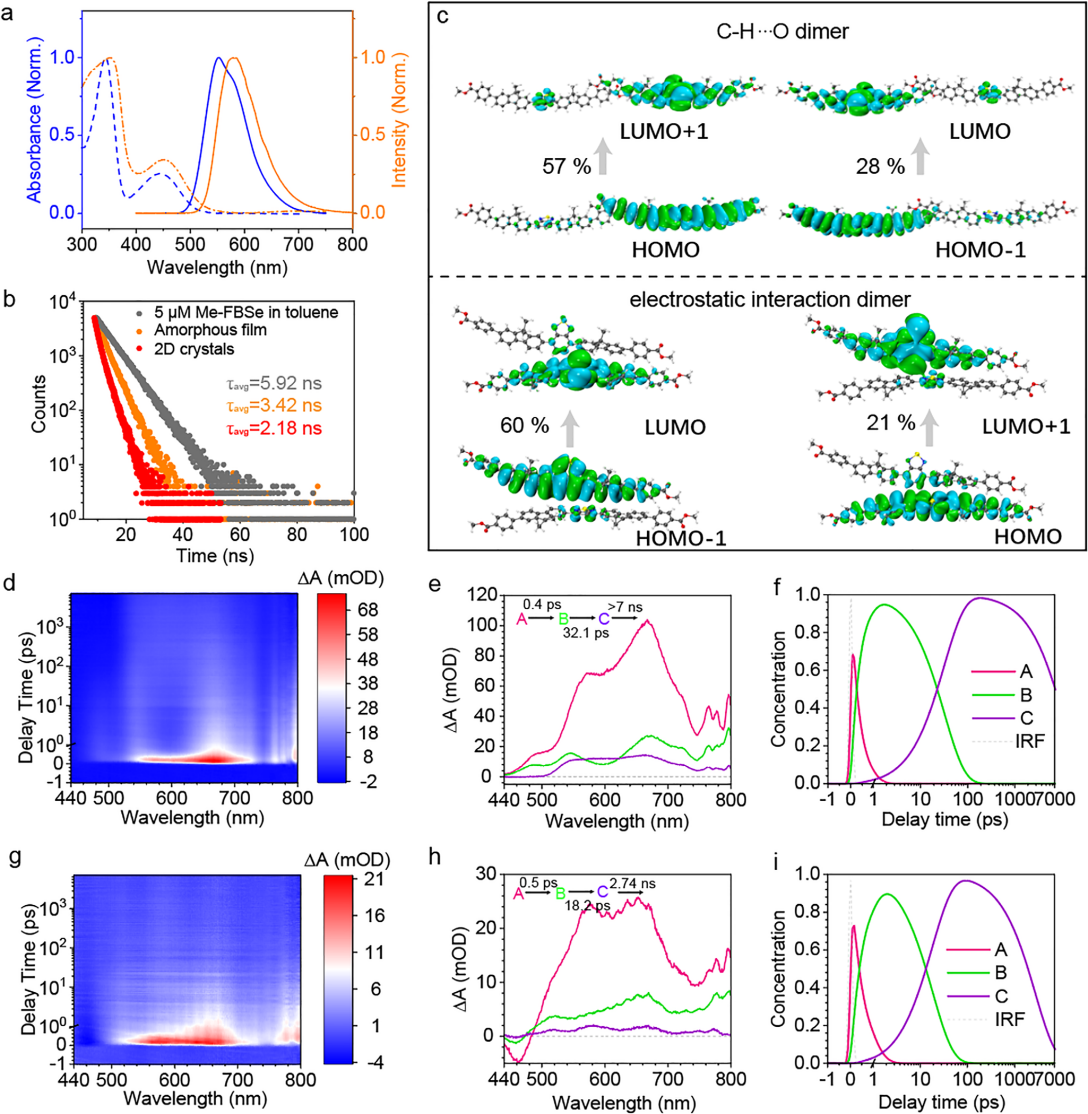
The excited‐state analysis of 2D crystals of **Me‐FBSe**. (a) Normalized absorption spectra of **Me‐FBSe** in toluene (dashed blue line) and 2D crystals of **Me‐FBSe** (dashed orange line) and normalized fluorescence spectra of **Me‐FBSe** in toluene (solid blue line) and 2D crystals of **Me‐FBSe** (solid orange line); (b) Time‐resolved PL spectra of **Me‐FBSe** in toluene, amorphous film of **Me‐FBSe**, and 2D crystals of **Me‐FBSe**; (c) Orbital transitions: C─H···O dimer (up) and electrostatic interaction dimer (bottom) of **Me‐FBSe**; Two‐dimensional plots of the TA spectra of 2D crystals of **Me‐FBSe** (d) and amorphous film of **Me‐FBSe** (g) were pumped at 365 nm; SADS (e) and transient component evolution (f) of 2D crystals of **Me‐FBSe**; SADS (h) and transient component evolution (i) of amorphous film of **Me‐FBSe**.

First, the absorption and fluorescence spectra of the 2D crystals of **Me‐FBSe** were compared with those of a 5 µM solution of **Me‐FBSe** in toluene. As shown in Figure 3a, a redshift was observed in the charge‐transfer absorption band from 445 nm to 453 nm. The fluorescence emission peak of the 2D crystals of **Me‐FBSe** is red‐shifted from 553 nm to 580 nm, which is almost identical to that of the 3D crystals of **Me‐FBSe** at 582 nm (Figure S22), suggesting that the 2D crystals and 3D crystals possess similar molecular packing. Then, the relatively weak electronic coupling in the 2D crystals of **Me‐FBSe** allows it to maintain higher emission efficiency, with a photoluminescence quantum yield (PLQY) of approximately 36%. In comparison, the amorphous film of **Me‐FBSe** was fabricated via optimized spin‐coating processes, with a thickness of approximately 24–50 nm (Figure S23), and exhibited a lower PLQY of 29%. Furthermore, time‐resolved photoluminescence spectroscopy was conducted to obtain more insight into the emission dynamics (Figure 3b). The 5 µM solution of **Me‐FBSe** in toluene exhibited a single exponent decay with time constants of τ = 5.92 ns. While the spin‐coated film exhibited two exponent decay with time constants of τ_1_ = 3.74 ns (85.94%) and τ_2_ = 1.45 ns (14.06%). The calculated average time of τ_avg_ = 3.42 ns exhibits a shorter fluorescence lifetime, which confirms the interaction between **Me‐FBSe** in spin‐coated amorphous film. Importantly, the emission decay is faster in the 2D crystals of **Me‐FBSe**, exhibiting a two fast decay progress with time constants of τ_1_ = 2.70 ns (67.82%) and τ_2_ = 1.08 ns (32.18%), and the calculated average time of τ_avg_ was about 2.18 ns. The results above demonstrate that 2D crystals of **Me‐FBSe** exhibit higher photoluminescence quantum yields and shorter lifetimes compared to amorphous films. As shown in Table S2, both the radiative decay rate constants *k*_r_ and non‐radiative decay rate constants *k*_nr_ are larger in the 2D crystals than in the amorphous film. Specifically, *k*_r_ increases by approximately 1.95‐fold in the 2D crystals compared with the amorphous film, whereas *k*_nr_ increases by approximately 1.42‐fold. As a result, the radiative decay channel becomes more competitive in the 2D crystals. The higher fluorescence quantum yield of the 2D crystals of **Me‐FBSe** is mainly attributed to the enhanced radiative decay rate. Besides, the oscillator strengths of **Me‐FBSe** in monomeric and dimeric configurations are also obtained from the time‐dependent density functional theory (TD‐DFT) calculations (Table S3 and Figure S24), demonstrating that the oscillator strength of **Me‐FBSe** is indeed enhanced under crystal‐constrained geometry, suggesting that the larger radiative rate constant (*k*_r_) of the 2D crystals is partly associated with increased radiative transition probability.

Next, to elucidate the influence of intermolecular interactions in 2D crystals of **Me‐FBSe** on excited states, TD‐DFT calculations at the single‐point level were performed on dimeric structures extracted from the **Me‐FBSe** single crystal. Within the structure of single crystal, four **Me‐FBSe** molecules are associated into tetrameric units via electrostatic interactions. Furthermore, these clusters are interconnected along the a and c crystallographic axes by weak intermolecular C─H···O interactions (Figure S16). As shown by the electron orbital distributions (Figure 3c), for the dimer stabilized by intracluster electrostatic interactions, the electronic transition involves intermolecular charge transfer (CT) between the two **Me‐FBSe** molecules. In contrast, for the relatively weak C─H···O dimer, the effective transitions are mainly localized with in the same **Me‐FBSe** molecule. This comparison suggests that the photoluminescence of the **Me‐FBSe** crystal is primarily governed by the electronic properties within the tightly bound electrostatic clusters. The weak C─H···O interaction between the clusters effectively decouples the adjacent emitting units. Therefore, the restricted intermolecular connection between the clusters promotes an efficient luminescence pathway that is largely confined to the tetrameric units, resulting in high emission efficiency in the solid state.

Then, transient absorption (TA) spectroscopy was used to reveal the excited‐state dynamics of **Me‐FBSe** in CHCl_3_, 2D crystals of **Me‐FBSe**, and amorphous film. As shown in Figure S25, in a medium‐polarity solvent chloroform, the initial excited state absorption (ESA) between 450 and 750 nm, recorded at 100 fs, is attributed to the locally excited (LE) state (A). This state undergoes ultrafast relaxation to a charge‐transfer (CT*) state (B), characterized by a blue‐shifted ESA at 580 nm, a red‐shifted and attenuated ESA signal at 680 nm, and a concurrent enhancement of ESA around 500 nm. These spectral changes indicate rapid LE‐to‐CT transition driven by solvation [47, 48]. Subsequently, a solvation‐stabilized CT state (C) is formed. The transformation from C to D occurs within 3.1 ns, marked by a decay of ESA at 520 nm and a rise at 600 nm, suggesting possible intersystem crossing facilitated by the presence of selenium atoms, leading to a long‐lived triplet state with a lifetime exceeding 7 ns, which is detrimental to both the material's fluorescence efficiency and photostability. In 2D crystals of **Me‐FBSe**, global target analysis of transient absorption data identifies three evolution steps (Figure 3d–f): an initial LE state (A) that converts within 0.4 ps into a CT state (B), which further relaxes in 32.1 ps to a stabilized relaxed CT state. This relaxed CT state exhibits extended longevity in the 2D crystals, remaining detectable beyond 7 ns. This long‐lived component suggests that ordered molecular packing stabilizes a CT‐associated excited‐state population through intracluster interactions. This behavior is consistent with the cluster‐isolated packing discussed above, which may enhance intracluster radiative transitions while suppressing strong intercluster electronic coupling. In contrast, within amorphous film, structural disorder promotes faster charge recombination, shortening the lifetime of the relaxed CT state to 2.74 ns (Figure 3g–i). Furthermore, the photostability of 2D crystals and amorphous films of **Me‐FBSe** under continuous irradiation was compared. As shown in Figure S26, the fluorescence intensity of the 2D crystals of **Me‐FBSe** decreased by only 1%, whereas that of the amorphous film decreased by 7% after 2 h of UV irradiation (385 nm, 10 mW/cm^2^) in air. These results indicate that the 2D crystals of **Me‐FBSe** exhibits improved photostability compared with the amorphous film.

### 2.5 Detection of DMS

**FIGURE 4:**
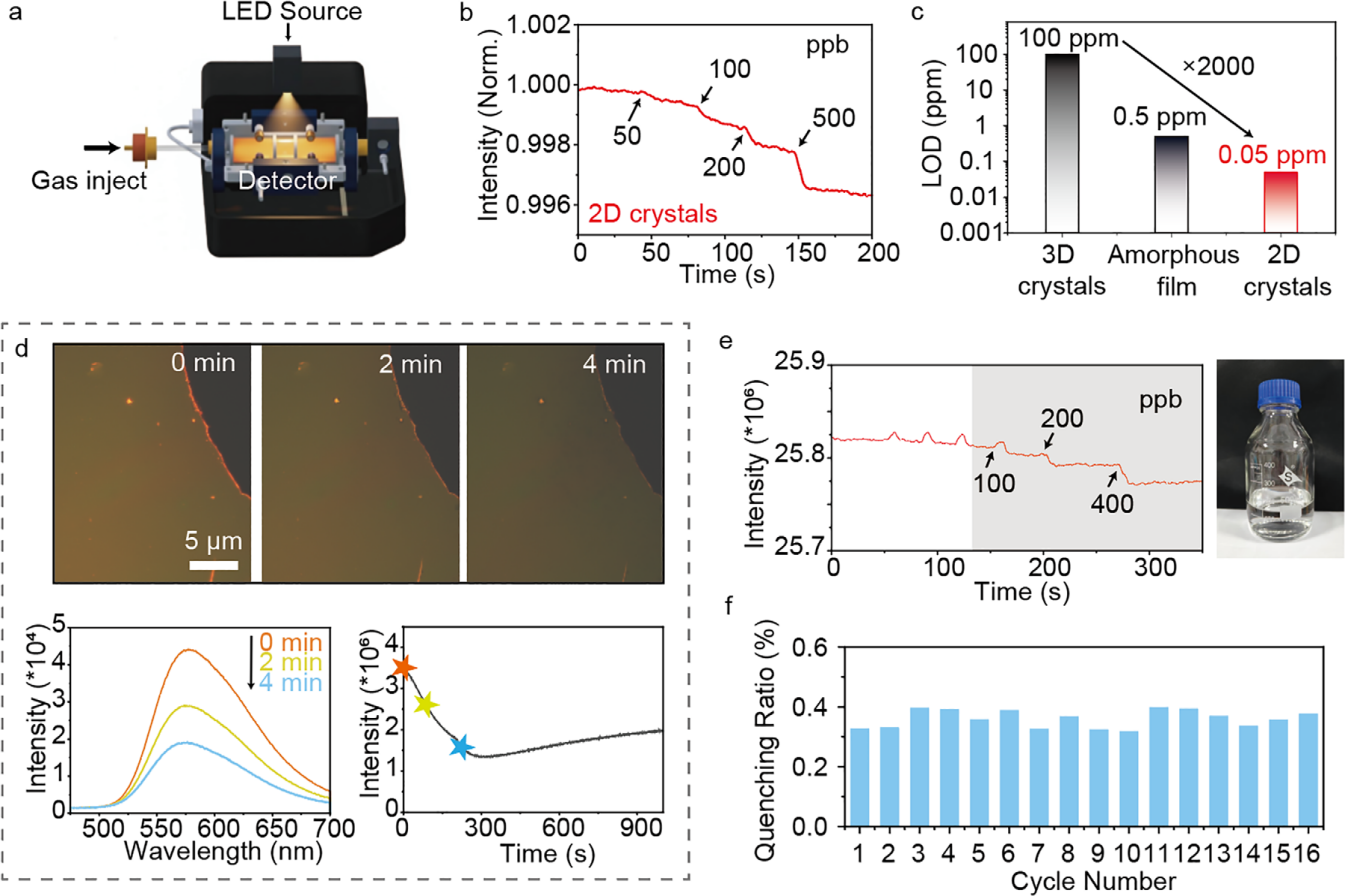
Selective recognition DMS for 2D crystals of **Me‐FBSe**. (a) Diagram of the fluorescent sensing device containing a glass tube with sensing materials coupled a built‐in silicon diode detector. (b) Real‐time fluorescence responses of 2D crystals of **Me‐FBSe** upon exposure to varying concentrations of DMS vapor. (c) Histogram of LOD for DMS detection using different forms of **Me‐FBSe**. (d) Synchronous response of the 2D crystals of **Me‐FBSe** to DMS vapor, illustrated by fluorescence microscopy images, emission spectra, and the corresponding time‐dependent response based on the integration of the fluorescence peak area. (e) Time‐dependent fluorescence responses of 2D crystals of **Me‐FBSe** upon exposure to headspace detection from varying concentrations of DMS in water, demonstrating a detection limit of 100 ppb. (f) Reusability test of the sensor over 16 cycles of exposure to 1 mg/L DMS in aqueous solution.

Having obtained the 2D crystals of **Me‐FBSe** with abundant active sites, high emission efficiency, we further explored its sensing performance toward DMS. The monitoring of DMS is critically important for understanding atmospheric climate feedback mechanisms, ensuring industrial safety, and enabling non‐invasive medical diagnostics [49, 50, 51, 52]. The fluorescence response of the material to DMS was evaluated using a home‐built portable sensing system (Figure 4a), which was integrated with a silicon photodiode detector. The 2D crystals of **Me‐FBSe** demonstrated a significant fluorescence response to trace DMS from 50 to 500 ppb (Figure 4b). Furthermore, the fluorescence quenching response was fast, taking 11 s for 50 ppb DMS vapor (Figure S27). To further validate the structural advantages, a disordered amorphous film was also fabricated as a control. As shown in Figure S28a, the quenching ratio of the 2D crystals was substantially larger than that of the amorphous film at equivalent DMS vapor concentrations like 500 ppb. In sharp contrast, the 3D **Me‐FBSe** crystals, despite sharing the same crystal structure, exhibited no detectable response until the concentration reached 100 ppm (Figure S28b). This superiority is highlighted by the limit of detection (LOD) analysis (Figure 4c), which reveals a 2000‐fold sensitivity enhancement for the 2D crystals over the 3D crystals. The superior performance of the 2D crystals of **Me‐FBSe** can be attributed to its high density of exposed active sites and high emission efficiency, which collectively enhance the sensing response. To directly visualize and characterize the sensing process of the 2D crystals of **Me‐FBSe**, we employed a multi‐modal in situ monitoring platform (Figure S29) integrating fluorescence microscopy, spectroscopy, and a gas reaction chamber. This system was used to visualize the fluorescence response and response dynamics of the 2D crystals of **Me‐FBSe** upon exposure to DMS vapor. As shown in Figure 4d, exposure to 5000 ppm DMS led to a pronounced fluorescence intensity decrease of the 2D crystals, enabling the quenching process to be directly observed under a fluorescence microscopy. The fluorescence micrographs show an overall decrease in emission across the observed region, indicating that the 2D crystals of **Me‐FBSe** exhibit a clear visual response to DMS vapor under high‐concentration conditions. This spatial observation is consistent with the decrease in the corresponding fluorescence emission intensity and the time‐dependent quenching profile.

Moreover, the 2D crystals of **Me‐FBSe** demonstrate exceptional selectivity for DMS vapor over other potential interferents, like water vapor, and common organic solvents, as evidenced in Figure S30, where thioether compounds triggered a distinct fluorescence quenching, whereas other substances exhibited fluorescence enhancement, none exhibited the high sensitivity observed for DMS. Theoretical calculations in Figure S31 support this high selectivity, suggesting that the sulfur atom in DMS possesses the highest relative electronegativity among the tested sulfides, which may be the key determinant for the observed ultra‐sensitive detection. Remarkably, the sensor could also effectively detect DMS in aqueous solution with a limit of detection as low as 100 ppb as shown in Figure 4e. Subsequent reusability tests revealed that the sensing performance of the 2D crystals of **Me‐FBSe** remained stable upon repeated exposure to 1 mg/L of DMS in aqueous solution over 16 cycles in Figure 4f and Figure S32. So, the 2D crystals of **Me‐FBSe** exhibited excellent durability, maintaining its high detection performance across multiple testing cycles, which underscores its significant potential for practical, long‐term sensing applications.

### 2.6 Sensing Mechanism

**FIGURE 5:**
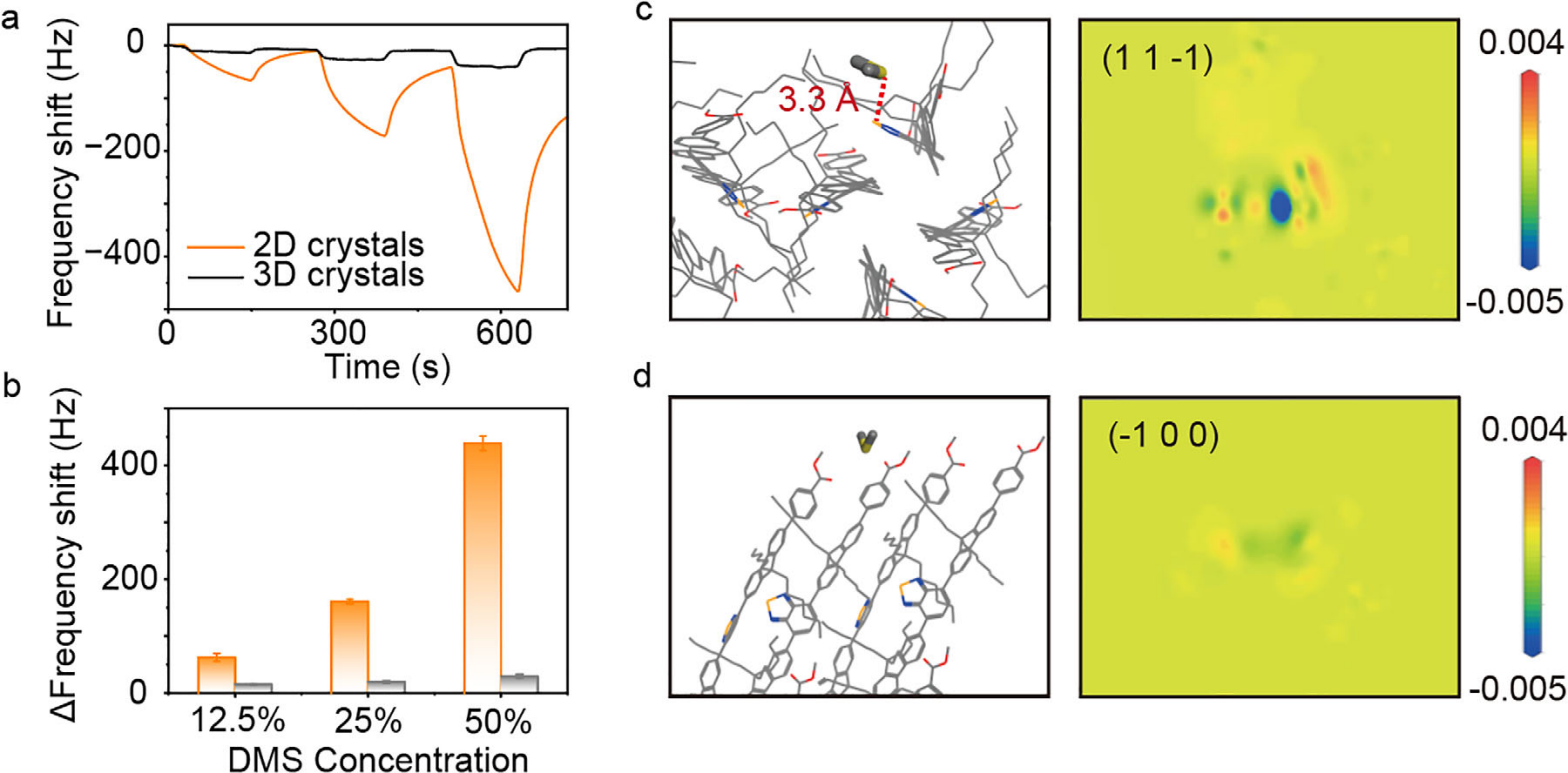
Investigation of the sensing mechanism and binding affinity. (a) Real‐time QCM frequency responses of 2D crystals and 3D crystals of **Me‐FBSe** upon exposure to DMS vapor. (b) Histogram of frequency changes under 12.5%, 25%, and 50% DMS concentrations. (c, d) DFT‐calculated interaction mechanisms of DMS with the (1 1 ‐1) and (‐1 0 0) facets of **Me‐FBSe** crystals.

The ultra‐sensitive sensing capability of the 2D crystals of **Me‐FBSe** toward DMS vapor was compared with that of 3D crystals using a QCM system. As shown in Figure 5a, the 2D crystals of **Me‐FBSe** exhibited a significantly larger frequency shift than the 3D crystals at the same DMS concentration, indicating the superior binding affinity of DMS to the 2D crystals. As the frequency shifts increase with the concentration ranging from 12.5% to 50% (balance N_2_), the difference between the adsorptions of DMS also became large (Figure 5b). Density functional theory (DFT) calculations were performed to gain deeper insights into the interactions between the DMS and different facet. Based on XRD analysis, the (1 1 ‐1) facet was identified as the primary exposed surface of 2D crystals. On this facet, the Chalcogen bonding is evidenced by a Se···S distance of 3.3 Å. Furthermore, the calculated binding energy on the (1 1 ‐1) facet is 28.9 kcal mol^−1^ (Figure 5c). In sharp contrast, the binding energy on the (‐1 0 0) facet is significantly lower at 14.6 kcal mol^−1^ (Figure 5d). These findings demonstrate that the sufficient exposure of benzoselenadiazole active sites on the (1 1 ‐1) facet is critical for enhancing the recognition of target DMS molecules, thereby simultaneously maximizing detection sensitivity and selectivity.

## 3 Conclusions

In this work, a donor‐acceptor‐donor (D‐A‐D) fluorophore, **Me‐FBSe**, with high photoluminescence efficiency was synthesized featuring terminal methyl ester groups. The solvent‐water interface self‐assembly strategy was employed to fabricate 2D crystals of **Me‐FBSe**. This strategy effectively overcame the limitation of recognition sites being encapsulated in conventional 3D crystals, leading to the sufficient exposure of the benzoselenadiazole recognition groups, which is crucial for sensing applications. Transient absorption and fluorescence spectroscopy confirmed the presence of a localized charge transfer (CT) excited state within the 2D crystals of **Me‐FBSe**. The ordered packing of 2D crystals also facilitates an increase in the radiative decay rate, thus endowing the material with high intrinsic fluorescence properties. Leveraging the material's high photoluminescence quantum yield and the sufficient exposed active recognition sites, the 2D crystals of **Me‐FBSe** sensing material was integrated into a coaxial gas chamber equipped with a silicon photodetector. This integrated system demonstrated high‐sensitivity detection of the organic sulfur compound DMS, and achieved the capability for rapid assessment of DMS pollution in aqueous solutions. This work provides a new avenue for designing and developing high‐performance and ultra‐sensitive organic sulfur sensing materials and devices.

## 4 Experimental Methods

The synthesis of fluorophore **Me‐FBSe**, fabrication of 3D crystals, 2D crystals, and amorphous film of **Me‐FBSe**, property characterizations, theoretical calculations, Figures S14‐S32, and Tables S1‐S3 are described in the Supporting Information.

## Author Contributions

**Yanjun Gong**: conceptualization, writing – review and editing, writing – original draft, funding acquisition, visualization. **Shuya Liu**: data curation, conceptualization, funding acquisition, visualization, formal analysis, validation, writing – original draft, writing – review and editing. **Chuanqin Cheng**: methodology, data curation. **Yueqiang Zhang**: formal analysis, data curation. **Jin Wang**: writing – review and editing, supervision. **Yonglei Wang**: writing – original draft, writing – review and editing. **Feiyong Chen**: funding acquisition.

## Funding

This work was supported by the Natural Science Foundation of Shandong Province (Nos. ZR2025QC1389, and ZR2024YQ012), National Natural Science Foundation of China (No. 22522606), Shandong Top Talent Special Foundation‐Research and Development of Key Technologies and High‐End Equipment of Water Environment Health in the Yellow River Basin (Shandong) (Granted number No. 0031504), and the Instrumentation and Equipment Capability Enhancement Project of Shandong University (No. ts20240101).

## Conflicts of Interest

The authors declare no conflicts of interest.

## Supporting information




**Supporting File**: advs76473‐sup‐0001‐SuppMat.docx.

## Data Availability

The data that supports the findings of this study are available in the supplementary material of this article.
