# Supplementary material for: Interface‐Directed High Fluorescence Efficiency Two‐Dimensional Molecular Crystals With Surface‐Exposed Active Sites
Source: Adv Sci (Weinh). 2026 Jul 9;13(54):e76473. doi: 10.1002/advs.76473 (PMC13348653; doi:10.1002/advs.76473)
Supplement: Supplementary file 1 [file ADVS-13-e76473-s001.docx]

Supporting Information

Interface-Directed High Fluorescence Efficiency Two-Dimensional Molecular Crystals with Surface-Exposed Active Sites

*Shuya Liu^a,b^, Yueqiang Zhang^c^, Chuanqin Cheng^d^*, *Jin Wang^a,b,^*, Feiyong Chen^a,b^, Yonglei Wang ^a,b,^*, and Yanjun Gong^c,^**

Shuya Liu, Jin Wang, Feiyong Chen, Yonglei Wang,

Institute of Resources and Environment Innovation, Shandong Jianzhu University, Jinan 250101, Shandong, China.

School of Municipal and Environmental Engineering, Shandong Jianzhu University, Jinan 250101, Shandong, China.

E-mail: wangjin21@sdjzu.edu.cn; wyl1016@sdjzu.edu.cn,

Yueqiang Zhang, Yanjun Gong

School of Chemistry and Chemical Engineering, Shandong University, Jinan, Shandong 250100, China.

E-mail: yjgong@sdu.edu.cn

Chuanqin Cheng

Key Laboratory of Organic Optoelectronics and Molecular Engineering of Ministry of Education, Department of Chemistry, Tsinghua University, Beijing 100084, P. R. China

**Table of Contents**

1. Synthesis of **Me-FBSe**

2. Fabrication of 3D crystals, 2D crystals, and amorphous film of **Me-FBSe**

3. Property characterizations

4. Theoretical calculations

5. Figures S14 to S32 and Tables S1 to S3

6. References

1. **Synthesis of Me-FBSe**

**Scheme S1.** Synthesis procedures of **Me-FBSe**.

**Methyl 4-(7-bromo-9,9-dihexyl-9H-fluoren-2-yl) benzoate (5)**. The solution of (4-(methoxycarbonyl) phenyl) boronic acid (0.70 g, 3.89 mmol), 2,7-dibromo-9,9-dihexyl-9H-fluorene (2.30 g, 4.67 mmol), and tetrakis(triphenylphosphine)palladium (225 mg, 0.195 mmol) in deoxygenated 1,4-dioxane (30 mL) was stirred, and an aqueous solution of potassium carbonate (6 mL, 2.0 M) was added. The mixture was then heated to 80°C and stirred overnight under an argon atmosphere. After the reaction, the solvent was removed under vacuum, and the residue was poured into water (50 mL) and extracted with ethyl acetate (3 × 50 mL). The combined organic layers were washed with saturated brine, dried over Na_2_SO_4_, and concentrated under reduced pressure. The residue was purified by column chromatography on silica gel using a petroleum ether: ethyl acetate (20:1) mixture as the eluent, yielding **5** (1.53 g, 2.80 mmol, 72% yield).

**fluorophore 5.** ^1^H NMR (400 MHz, CDCl_3_) δ 8.14-8.12 (d, J = 8.0 Hz, 2H), 7.74-7.71 (m, 3H), 7.61-7.56 (m, 3H), 7.48-7.46 (m, 2H), 3.95 (s, 3H), 2.05-1.92 (m, 4H), 1.14-1.09 (m, 4H), 1.07-1.03 (m, 6H), 0.89-0.83 (m, 2H), 0.78-0.74 (t, J = 16.0 Hz, 6H), 0.69-0.61 (m, 4H)

^13^C NMR (101 MHz, CDCl_3_) δ 167.00, 153.32, 151.25, 145.91, 140.25, 139.55, 139.26, 130.17, 130.14, 128.84, 127.07, 126.46, 126.27, 121.58, 121.44, 121.30, 120.24, 55.61, 52.14, 40.30, 31.47, 29.64, 23.76, 22.59, 14.02.

ESI⁺ HRMS: m/z 549.2191 ([M + 2H]⁺, calcd 549.57)

**Figure S1.** ^1^H NMR of fluorophore **5**.

**Figure S2.** ^13^C NMR of fluorophore **5.**

**Figure S3.** MS-ESI of fluorophore **5**.

**Methyl 4-(9,9-dihexyl-7-(4,4,5,5-tetramethyl-1,3,2-dioxaborolan-2-yl)-9H-fluoren-2-yl) benzoate (4).** A mixture of methyl 4-(7-bromo-9,9-dihexyl-9H-fluoren-2-yl)benzoate **5** (1.53 g, 2.80 mmol), 4,4,4',4',5,5,5',5'-octamethyl-2,2'-bi(1,3,2-dioxaborolane) (1.07 g, 4.20 mmol), potassium acetate (0.82 g, 8.40 mmol), and [1,1'-bis(diphenylphosphino)ferrocene]dichloropalladium(II) (Pd(dppf)Cl₂, 0.10 g, 0.14 mmol) in anhydrous 1,4-dioxane (25 mL) was degassed and purged with argon three times. The reaction mixture was then heated to 85 °C and stirred for 12 hours under an argon atmosphere. After cooling to room temperature, the solvent was removed under reduced pressure. The residue was poured into water (50 mL) and extracted with dichloromethane (3 × 50 mL). The combined organic layers were washed with saturated brine, dried over anhydrous sodium sulfate, and concentrated under vacuum. The crude product was purified by column chromatography on silica gel using a mixture of petroleum ether and ethyl acetate (15:1, v/v) as the eluent to afford the title compound **4** as a white solid (1.43 g, 80% yield).

**fluorophore 4.** ^1^H NMR (400 MHz, CDCl_3_) δ 8.14-8.12 (d, J = 8.0 Hz, 2H), 7.84-7.82 (d, J = 8.0 Hz 1H), 7.81-7.79 (d, J = 8.0 Hz 1H), 7.77 (s, 1H), 7.74-7.72 (d, J = 8.0 Hz 3H), 7.62-7.60 (d, J = 8.0 Hz 1H), 7.58 (s, 1H), 3.95 (s, 3H), 2.06-1.99 (m, 4H), 1.40 (s, 12H), 1.11-1.00 (m, 12H), 0.76-0.73 (t, J = 12.0 Hz, 6H), 0.67-0.59 (m, 4H)

^13^C NMR (101 MHz, CDCl_3_) δ 167.07, 152.19, 150.25, 146.14, 143.47, 141.18, 139.21, 133.86, 130.12, 128.92, 128.71, 127.09, 126.20, 121.64, 120.52, 119.22, 83.77, 55.32, 52.13, 40.23, 31.44, 29.64, 24.96, 23.70, 22.56, 14.00.

ESI⁺ HRMS: m/z 595.3953 ([M + H]⁺, calcd 595.64)

**Figure S4.** ^1^H NMR of fluorophore **4**.

**Figure S5.** ^13^C NMR of fluorophore **4.**

**Figure S6.** MS-ESI of fluorophore **4**.

**Dimethyl 4,4'-(benzo[c] [1,2,5] thiadiazole-4,7-diylbis(9,9-dihexyl-9H-fluorene-7,2-diyl)) dibenzoate (3).** A solution of methyl 4-(9,9-dihexyl-7-(4,4,5,5-tetramethyl-1,3,2-dioxaborolan-2-yl)-9H-fluoren-2-yl)benzoate (1.43 g, 2.24 mmol), 4,7-dibromobenzo[c][1,2,5]thiadiazole (0.29 g, 0.98 mmol), and tetrakis(triphenylphosphine)palladium (80 mg, 0.07 mmol) in degassed 1,4-dioxane (20 mL) was stirred. An aqueous solution of potassium carbonate (4 mL, 2.0 M) was then added. The mixture was heated to 80 °C and stirred for 12 hours under an argon atmosphere. After the reaction was complete, the solvent was removed under reduced pressure. The residue was poured into water (60 mL) and extracted with dichloromethane (3 × 50 mL). The combined organic layers were washed with saturated brine, dried over anhydrous sodium sulfate, and concentrated under vacuum. The crude product was purified by column chromatography on silica gel using a mixture of petroleum ether and dichloromethane (1:1, v/v) as the eluent to afford the title compound **3** as an orange solid (1.24 g, 78% yield).

**fluorophore 3.** ^1^H NMR (400 MHz, CDCl_3_) δ 8.16-8.14 (d, J = 8.0 Hz, 4H), 8.07-8.05 (d, J = 8.0 Hz, 2H), 8.0 (s, 2H), 7.92-7.90 (m, 4H), 7.87-7.85 (d, J = 8.0 Hz, 2H), 7.77-7.75 (d, J = 8.0 Hz, 4H), 7.67-7.64 (m, 4H), 3.97 (s, 6H), 2.17-2.04 (m, 8H), 1.17-1.06 (m, 24H), 0.86-0.82 (m, 8H), 0.79-0.75 (t, J = 16.0 Hz, 12H)

^13^C NMR (101 MHz, CDCl_3_) δ 167.08, 154.38, 152.24, 151.50, 146.11, 140.90, 140.73, 139.04, 136.54, 133.60, 130.16, 128.74, 128.33, 127.96, 127.10, 126.41, 124.05, 121.69, 120.40, 120.02, 55.46, 52.16, 40.33, 31.50, 29.74, 23.93, 22.60, 14.04.

ESI⁺ HRMS: m/z 1069.5727, ([M]⁺, calcd 1069.50)

**Figure S7.** ^1^H NMR of fluorophore **3.**

**Figure S8.** ^13^C NMR of fluorophore **3.**

**Figure S9.** MS-ESI of fluorophore **3**.

**Dimethyl 4,4’-(5,6-diaminocyclohexa-1,3-diene-1,4-diyl) bis(9,9-dihexyl-9H-fluorene-7,2-diyl)) dibenzoate (2).** Sodium borohydride (340 mg, 9.0 mmol) was added to a solution of dimethyl 4,4'-(benzo[c] [1,2,5] thiadiazole-4,7-diylbis(9,9-dihexyl-9H-fluorene-7,2-diyl)) dibenzoate **3** (1.24 g, 0.90 mmol) in a mixture of ethanol (EtOH, 30 mL) and tetrahydrofuran (THF, 25 mL) at 0 °C. The reaction mixture was then refluxed until the yellow color (or fluorescence) in the solution disappeared. After the solvent was removed under reduced pressure, water (50 mL) was added, and the mixture was extracted with dichloromethane (3 × 30 mL). The combined organic layers were washed with brine, dried over anhydrous Na₂SO₄, and concentrated under vacuum to yield compound **2** as a crude product. Compound **2** was used directly in the next step without further purification.

**Dimethyl 4,4'-(benzo[c] [1,2,5] selenadiazole-4,7-diylbis(9,9-dihexyl-9H-fluorene-7,2-diyl)) dibenzoate (1，Me-FBSe).** To a solution of the crude compound **2** (from the previous step, assumed ~0.90 mmol) in ethanol (30 mL) and tetrahydrofuran (25 mL), selenium dioxide (400 mg, 3.60 mmol) dissolved in hot water (5 mL) was added. The mixture was stirred at 25 °C for 2 hours. After removing the solvents under reduced pressure, the residue was poured into water (60 mL) and extracted with dichloromethane (3 × 50 mL). The combined organic layers were washed with saturated brine, dried over anhydrous Na₂SO₄, and concentrated under vacuum. The crude product was purified by column chromatography on silica gel using a mixture of petroleum ether and dichloromethane (1:1, v/v) as the eluent, yielding compound **Me-FBSe** as a solid (0.60 g, 41% yield over two steps from compound **3**). The resulting target compound **Me-FBSe** was confirmed by ^1^H NMR,^13^C NMR, MALDI-MS as well as COSY, below.

**Me-FBSe.** ^1^H NMR (400 MHz, CDCl_3_) δ 8.15 (m, 4 H), 8.00 (dd, J = 8.0, 1.6 Hz, 2 H), 7.90 (d, J = 8.0, 2 H), 7.89 (s, 2 H), 7.85 (d, J = 7.8 Hz, 2 H), 7.76 (m, 4 H), 7.75 (s, 2 H), 7.66 (dd, J = 7.8, 1.6 Hz, 2 H), 7.64 (s, 2 H), 3.97 (s, 6 H) 2.09 (m, 8 H), 1.12 (m, 24 H), 0.83 (m, 8 H), 0.77 (t, J = 6.8 Hz, 12 H).

^13^C NMR (101 MHz, CDCl3) δ 167.23, 160.14, 152.34, 151.51, 146.26, 141.07, 140.73, 139.11, 137.30, 135.53, 130.29, 128.84, 128.79, 128.50, 127.23, 126.53, 124.37, 121.81, 120.49, 119.96, 55.55, 52.29, 40.44, 31.63, 29.87, 24.06, 22.73, 14.17. MALDI-MS: (m/z) = 1118.445.

**Figure S10.** ^1^H NMR of fluorophore **Me-FBSe**.

**Figure S11.** ^13^C NMR spectrum of fluorophore **Me-FBSe**.

**Figure S12.** MALDI-MS of fluorophore **Me-FBSe**.

**Figure S13.** H-H COSY spectrum of fluorophore **Me-FBSe**.

**2. Fabrication of 3D crystals, 2D crystals, and amorphous film of Me-FBSe.**

**2.1 Self-assembly of the 3D crystals of fluorophore Me-FBSe.**

3D crystals of fluorophore **Me-FBSe** were synthesized by adding 100 μL of a 0.8 mM chloroform solution of **Me-FBSe** to 1 mL ethanol, followed by aging at 25 °C for 7 d.

**2.2 Preparation of 2D crystals of Me-FBSe via interface assembled method**

A 100 μL of chloroform solution of **Me-FBSe** (1-10 mg/mL) was slowly added dropwise onto the surface of 3 mL of water in a 5 mL vial with 20 mm inner diameter. The entire apparatus was maintained at 4 ℃ to ensure the slow evaporation of the chloroform. As the solvent evaporated, the **Me-FBSe** self-assembled and crystallized at the interface. After the complete evaporation of chloroform, the film floating on the water surface was collected for subsequent characterization.

**2.3 Preparation of amorphous film of Me-FBSe by spin-coating**

An amorphous film of **Me-FBSe** was prepared by spin-coating. A 100 μL of the **Me-FBSe** solution in chloroform (10 mg/mL) was dynamically dispensed onto the center of a quartz substrate already rotating at 2000 rpm. The substrate was kept spinning at this speed for 90 s until the solvent fully evaporated.

**3. Property characterizations**

**3.1 Materials**

All chemicals were purchased from commercial suppliers and used without further purification.

**3.2 Characterizations of Me-FBSe monomers and 3D crystals, 2D crystals, and amorphous film by Me-FBSe structure and properties**

^1^H NMR, ^13^C NMR, and COSY spectra were recorded with a BRUKER AVANCE NEO 400M.

MS spectra were with a BRUKER Impact II.

Fluorescence-mode optical microscopic images were acquired using an Olympus IX71 fluorescence microscope with a standard CCD detector, in which the samples were excited by a 365 nm ultraviolet LED lamp. Bright-field micrographs were acquired on an Olympus microscope.

SCXRD analysis was performed on a Bruker D8 VENTURE diffractometer with graphite-monochromated Mo Kα radiation (λ = 0.71073 Å) in ω-scan mode.

Powder X-Ray diffraction analysis was carried out with a Rigaku model SmartLab X-ray diffractometer with monochromated CuKα radiation (λ = 1.5418 Å) at room temperature.

Static water contact angle (WCA) measurements were performed on a Drop Shape Analyzer. A 5 μL deionized water droplet was deposited onto the surface of the 2D crystals of **Me-FBSe** supported on a silicon wafer, and the contact angle was determined by analyzing the droplet profile with the instrument’s software.

Atomic force microscopy (AFM, Bruker Dimension Icon) was used to investigate the morphology of the sample. The 2D crystals of **Me-FBSe** were transferred to a silicon substrate.

UV-Vis absorption spectrum of a 5 μM solution of **Me-FBSe** in toluene were obtained on a Hitachi U-3900 spectrometer.

UV-Vis spectra of 2D crystals were obtained using UV-Visible-NIR microscope (CRAIC Technologies, Inc.). The samples were deposited onto a glass substrate, which was excited by 1.5 μm incident light for the collection of UV-Vis spectra.

The time-dependent fluorescence profiles were obtained by using an optical fiber connected to Ocean Optics USB4000 fluorometer to collect the emission intensity and using a 385 nm LED lamp (Ocean Optics, total output power 3 mW) coupled with an optical fiber as the light source (0.053 mW/cm^2^).

Selected area electron diffraction (SAED) pattern were acquired using JEOL 2100 with an electron beam energy of 120 kV. 2D crystals of **Me-FBSe** were deposited on copper grids for TEM characterizations.

Fluorescence quantum yields and fluorescence lifetimes were determined using an Edinburgh Instruments steady-state/transient fluorescence spectrometer coupled with an integrating sphere with a 365 nm pulsed laser serving as the excitation source.

PL spectra of a 5 μM solution of **Me-FBSe** in toluene and 2D crystals were measured with a Hitachi F-4700.

Scanning electron microscope (SEM) images were obtained on a Hitachi S-8010 field-emission microscope where the accelerating voltage and the current were set as 10 kV and 10 μA, respectively.

Femtosecond transient absorption (TAS) spectra were recorded on a Helios spectrometer (Coherent) coupled with an Astrella Ti:Sapphire laser system (Coherent, 1000 Hz, 100 fs). The pump pulse (365 nm) was generated by an optical parametric amplifier (OPerA Solo, Coherent), with its intensity adjusted by a variable neutral density filter. The probe pulse consisted of a white-light continuum (440-800 nm) generated in a sapphire crystal. The maximum pump-probe delay time was 7.6 ns, and the overall instrument response function (IRF) was approximately 140 fs. The excitation pulse energy was monitored with a PM400 power meter (Thorlabs).

Quartz crystal microbalance (QCM) measurements were performed using a conventional quartz crystal microbalance analyzer manufactured by Beijing Boinst Technology Co., Ltd.

**3.3 Sensing Experiments.**

The fluorescence sensing measurements of DMS vapor, H_2_O vapor, and other organic solvent vapors were performed using a portable fluorescence sensing system equipped with a silicon photodiode detector. By slowly evaporating chloroform solutions containing **Me-FBSe** at varying concentrations, two-dimensional **Me-FBSe** crystals were deposited in situ onto quartz tubes. Real-time fluorescence response was monitored by blowing 10 mL of the vapor of interest at a certain concentration into the chamber and the vapor was pumped into the chamber containing the sensing materials by an air pump (150 mL/min).

**4. Theoretical calculations.**

Initial molecular geometries were derived from single-crystal structure data. To simplify the model, the C6 alkyl chains were truncated to C1 (methyl) groups, and the structures were subsequently re-hydrogenated. Adiabatic excitation calculations for this monomer and dimer were performed using time-dependent DFT (TD-DFT) at the CAM-B3LYP/6-311G** level. The nosymm keyword was employed to prevent automatic re-orientation. This included geometry optimizations and frequency analyses for both the ground (S_0_) and excited (S_1_) states. All calculations were performed using the Gaussian 09 software package^[1]^. The resulting output files were analyzed using the Multiwfn program^[2]^. Molecular visualizations (e.g., orbitals, densities) were rendered using VMD^[3]^, with the isosurface value set to 0.005.

First-principles density functional theory (DFT) calculations were performed to investigate the binding energetics and electronic interactions between DMS and the (1 1 -1), (-1 0 0) crystal facets. All DFT calculations were conducted within the CASTEP module. The exchange-correlation interactions were modeled using the Perdew–Burke–Ernzerhof (PBE) functional under the generalized gradient approximation (GGA). A Monkhorst-Pack k-point grid of 2×2×2 was utilized for Brillouin zone integration, and the energy convergence threshold was rigorously set to 10^–5^ eV per atom to ensure computational accuracy.

**MD simulations.**

Molecular dynamics (MD) simulations were performed using the GROMACS package^[4]^. The system consisted of 10 **Me-FBSe** molecules, and 26,1586 water molecules. The initial configuration of the **Me-FBSe** and interfaces was generated using Packmol software^[5]^, showing **Me-FBSe** molecules were positioned at the center of the simulation box. The system was placed in a simulation box with dimensions of 10.16 nm×10.16×30.15 nm.

The force fields for **Me-FBSe** were generated using Sobtop software^[6]^, employing the Generalized AMBER Force Field (GAFF)^[7]^ and RESP charges, while the force field for water molecules was defined using the SPC/E model. During the simulation, the position of water remained stable throughout the entire simulation. The system was initially heated to 298.15 K, followed by energy minimization. The MD simulation was then performed in an NVT ensemble for a total simulation time of 12 ns.

In the NVT ensemble, temperature control was maintained using the V-rescale thermostat at 298.15 K. The LINCS algorithm was applied to constrain bond lengths of hydrogen atoms. Electrostatic interactions were evaluated using the Particle-Mesh Ewald (PME) method with a fourth-order interpolation, and a cutoff of 1.0 nm was used for the calculation of short-range van der Waals interactions.

**5. Figures S14 to S32 and Tables S1 to S3**

**Figure S14.** DFT-calculated (left) LUMO and HOMO values and (right) NTO analysis of **Me-FBSe**.

**Figure S15.** SEM image of 3D crystals of **Me-FBSe**.

**Figure S16.** The side-view single-crystal structure of **Me-FBSe** reveals the packing of clusters through weak interactions.

**Figure S17.** Dimensions and active sites of the different facets of 3D crystals of **Me-FBSe**, as derived from single-crystal structure and exposed facet orientation.

**Figure S18.** The photograph of 2D crystals of **Me-FBSe** on glass. Scale bar: 2.5 mm.

**Figure S19.** The SAED of 2D crystals of **Me-FBSe**.

**Figure S20.** Squared charge population analysis (SCPA) charge population analysis of **Me-FBSe** and H_2_O.

**Figure S21.** The AFM image, the 3D picture, and height profile of 2D crystals of **Me-FBSe** obtained by 10 mg/mL.

**Figure S22.** (a) Normalized fluorescence spectrum and (b) Time-resolved PL spectra of 3D crystals of **Me-FBSe.**

**Figure S23.** AFM height images and corresponding height profiles of the **Me-FBSe** amorphous film fabricated via spin-coating from a 10 mg mL^-1^ solution at 2000 rpm.

**Figure S24**. Calculated TD-DFT UV-vis spectra of monomer (orange) and dimer featuring the C-H···O packing interaction (red) of **Me-FBSe**.

**Figure S25**. (a) Two-dimensional plots of the TA spectra of **Me-FBSe** in chloroform were pumped at 365 nm; (b-c) TA spectra of **Me-FBSe** in chloroform evolved from the corresponding two-dimensional at the indicated time delays; SADS (d) and transient component evolution (e) of **Me-FBSe** in chloroform; (f) Decay kinetics fitting of **Me-FBSe** in chloroform probed at 520 nm, 600 nm, and 680 nm, respectively.

**Figure S26.** Fluorescence intensities of 2D crystals (orange line) and amorphous film (yellow line) as a function of UV irradiation time (385 nm, 10 mW/cm^2^) in air.

**Figure S27.** Response time of 2D crystals of **Me-FBSe** when exposed to 50 ppb DMS vapor.

**Figure S28.** Fluorescence responses of amorphous film (a) and 3D crystals (b) to DMS vapors at different concentrations.

**Figure S29.** The photo of a multi-modal in situ monitoring platform.

**Figure S30.** Fluorescence responses of 2D crystals of **Me-FBSe** to various interferents at different concentrations.

**Figure S31.** Electrostatic potential mapping diagram of different sulfides.

**Figure S32.** Time-dependent fluorescence response profiles of 2D crystals of **Me-FBSe** on exposure to 1 mg/L of DMS in water over 16 cycles.

**Table S1.** Crystallographic data and structural refinement summary of **Me-FBSe**.

| Compound | **Me-FBSe** |
| --- | --- |
| CCDC | Deposition Number 2518252 |
| Empirical formula | C72H80N2O4Se |
| Formula weight | 1116.34 |
| Temperature/K | 193.00 |
| Crystal system | monoclinic |
| Space group | P2_1_/c |
| a/Å | 23.4224(11) |
| b/Å | 23.2391(10) |
| c/Å | 23.2876(10) |
| α/° | 90 |
| β/° | 106.8350(10) |
| γ/° | 90 |
| Volume/Å^3^ | 12132.6(9) |
| Z | 8 |
| ρcalcg/cm^3^ | 1.222 |
| μ/mm^‑1^ | 0.670 |
| F(000) | 4736.0 |
| Crystal size/mm^3^ | 0.13 × 0.12 × 0.09 |
| Radiation | MoKα (λ = 0.71073) |
| 2Θ range for data collection/° | 3.948 to 50.818 |
| Index ranges | -28 ≤ h ≤ 28, -27 ≤ k ≤ 28, -28 ≤ l ≤ 28 |
| Reflections collected | 148563 |
| Independent reflections | 22222 [Rint = 0.1611, Rsigma = 0.0912] |
| Data/restraints/parameters | 22222/851/1658 |
| Goodness-of-fit on F2 | 1.018 |
| Final R indexes [I>=2σ (I)] | R1 = 0.0662, wR2 = 0.1428 |
| Final R indexes [all data] | R1 = 0.1456, wR2 = 0.1930 |
| Largest diff. peak/hole / e Å^-3^ | 0.71/-0.70 |

**Table S2.** Photophysical parameters of **Me-FBSe** in different states.

|  | λ_em_/ nm | ΦF | τ_avg_ / ns | *k*_r_ / 10^7^ s^-1^ | *k*_nr_ / 10^7^ s^-1^ |
| --- | --- | --- | --- | --- | --- |
| **Me-FBSe** in toluene | 553 | 0.90 | 5.92 | 15.2 | 1.69 |
| 2D crystals of **Me-FBSe** | 580 | 0.36 | 2.18 | 16.5 | 29.4 |
| amorphous film of **Me-FBSe** | 573 | 0.29 | 3.42 | 8.48 | 20.7 |
| 3D crystals of **Me-FBSe** | 582 | 0.33 | 3.66 | 9.02 | 18.3 |

**Table S3.** **Me-FBSe** TD-DFT Results, including Wavelengths (λ) and Oscillator Strengths (*f*).

|  | Wavelength (λ) | Oscillator Strength (*f*) |
| --- | --- | --- |
| the optimized monomer | 443.28 | 0.7981 |
| the monomer from crystal | 415.16 | 0.9188 |
| dimer featuring the C-H···O packing interaction | 415.98 | 1.9897 |

**References**

[1] Frisch, M.J., Trucks, G.W., Schlegel, H.B., Scuseria, G.E., Robb, M.A., Cheeseman, J.R., Scalmani, G., Barone, V., Mennucci, B., Petersson, G.A., Nakatsuji, H., Caricato, M., Li, X., Hratchian, H.P., Izmaylov, A.F., Bloino, J., Zheng, G., Sonnenberg, J.L., Hada, M., Ehara, M., Toyota, K., Fukuda, R., Hasegawa, J., Ishida, M., Nakajima, T., Honda, Y., Kitao, O., Nakai, H., Vreven, T., Montgomery Jr., J.A., Peralta, J.E., Ogliaro, F., Bearpark, M., Heyd, J.J., Brothers, E., Kudin, K.N., Staroverov, V.N., Kobayashi, R., Normand, J., Raghavachari, K., Rendell, A., Burant, J.C., Iyengar, S.S., Tomasi, J., Cossi, M., Rega, N., Millam, J.M., Klene, M., Knox, J.E., Cross, J.B., Bakken, V., Adamo, C., Jaramillo, J., Gomperts, R., Stratmann, R.E., Yazyev, O., Austin, A.J., Cammi, R., Pomelli, C., Ochterski, J.W., Martin, R.L., Morokuma, K., Zakrzewski, V.G., Voth, G.A., Salvador, P., Dannenberg, J.J., Dapprich, S., Daniels, A.D., Farkas, O., Foresman, J.B., Ortiz, J.V., Cioslowski, J., Fox, D.J., 2009. Gaussian 09, revision D.01. Gaussian, Inc, Wallingford, CT.

[2] Lu, T.; Chen, F., Multiwfn: a Multifunctional Wavefunction Analyzer. *J. Comput. Chem.* **2012**, *33*, 580-592.

[3] Humphrey, W.; Dalke, A.; Schulten, K., VMD: Visual Molecular Dynamics. *J. Mol. Graph.* **1996**, *14*, 33-38.

[4] Abraham, M. J.; Murtola, T.; Schulz, R.; Páll, S.; Smith, J. C.; Hess, B.; Lindahl, E. GROMACS: High performance molecular simulations through multi-level parallelism from laptops to supercomputers. *SoftwareX* **2015**, *1-2*, 19-25.

[5] Martínez, L.; Andrade, R.; Birgin, E. G.; Martínez, J. M., PACKMOL: a package for building initial configurations for molecular dynamics simulations. *J. Comput. Chem.* **2009**, *30*, 2157-2164.

[6] Lu, T., Sobtop, Version_1.0 (dev. 3.1), http://sobereva.com/soft/Sobtop (accessed on 5/18/2022)

[7] Wang, J.; Wolf, R. M.; Caldwell, J. W.; Kollman, P. A.; Case, D. A., Development and testing of a general amber force field. *J. Comput. Chem.* **2004**, *25*, 1157-1174.
